# STAT1 contributes to HLA class I upregulation and CTL reactivity after anti-EGFR mAb cetuximab therapy in head and neck cancer patients

**DOI:** 10.1186/2051-1426-1-S1-P175

**Published:** 2013-11-07

**Authors:** Raghvendra M Srivastava, Hyun-bae Jie, Soldano Ferrone, Robert L Ferris

**Affiliations:** 1 Department of Otolaryngology, University of Pittsburgh, Pittsburgh, PA, USA; 2 Department of Immunology, University of Pittsburgh, Pittsburgh, PA, USA; 3 Cancer Immunology Program, University of Pittsburgh, Pittsburgh, PA, USA; 4 Department of Surgery, Massachusetts General Hospital, Harvard Medical School, Boston, MA, USA

## 

Squamous cell carcinoma of head and neck (HNSCC) cells express low HLA class I and antigen processing machinery (APM) components, such as transporter TAP-1/2, which is associated with the reduced sensitivity to cytotoxic T lymphocyte (CTL) mediated lysis. Epidermal growth factor receptor (EGFR) is overexpressed in HNSCC and is associated with the poor prognosis. FDA approved anti-EGFR blockade mAb cetuximab inhibits HNSCC proliferation, and induces EGFR-specific CTL. However, the molecular mechanism(s) underlying the EGFR-specific CTL recognition of HNSCC in the therapeutic efficacy of anti-EGFR mAb is still emerging. We show that cetuximab or EGFR knockdown enhanced expression of HLA class I antigens, which is associated with the EGFR expression level on HNSCC. These findings were validated in a prospective trial of neoadjuvant cetuximab therapy. Interestingly, upregulation of HLA-B/C alleles were more pronounced than HLA-A alleles after cetuximab or EGFR knockdown treatment. EGFR signaling blockade or EGFR depletion also enhanced IFN gamma receptor (IFNAR) on HNSCC and augmented induction of HLA class I and TAP-1/2 caused by IFN gamma treatment. Cetuximab or EGFR knockdown enhanced the level of HLA class I, STAT-1, TAP-1/2 in a STAT-1+/+ cell line but not in STAT-1-/- cell line, documenting the STAT-1 dependence of this effect. We also found that Src homology domain-containing phosphatase 2 (SHP-2), which is downstream of EGFR and also overexpressed in SCCHN, can suppress the immunostimulatory effect of cetuximab treatment on HLA class I/STAT-1 upregulation, and dual targeting of EGFR and SHP-2 co-operates in the most efficient reversal of immune escape phenotype. In addition, cetuximab-based EGFR inhibition and SHP-2 depletion enhanced the recognition of HNSCC cells by EGFR _853-861_ specific CTL, and enhanced surface presentation of non-EGFR TA, such as MAGE-3 _271-279_ , indicating that a broad tumor antigen repertoire is processed and presented by HLA/APM upregulation. These findings elucidate a novel immune escape mechanism associated with EGFR signaling through STAT1 suppression and the reversal with cetuximab, which may provide additional targets for on-going mAb-based immunotherapy.

